# Assessing Subsoil Conditions with an ASABE Conform Vertical Penetrometer—Development and Evaluation

**DOI:** 10.3390/s23031306

**Published:** 2023-01-23

**Authors:** Oliver Schmittmann, Peter Schulze Lammers

**Affiliations:** Institut fuer Landtechnik, Universität Bonn, Nussallee 5, 53115 Bonn, Germany

**Keywords:** soil compaction, penetration resistance, shaft friction, heavy-duty penetrometer, calibration

## Abstract

Soil is the habitat for soil organisms and associated soil physical and chemical processes. The subsoil is a large reserve of water and nutrients. Soil and subsoil are thus significantly involved in the yield capacity of a site and its resilience in the case of unfavorable weather conditions. Subsoil can also retain water in drought phases and stores carbon. In times of climate change and scarcity of resources, many scientific activities involve subsoil and require sensors to assess subsoil conditions and properties. An electrically driven penetrometer with an integrated soil water content sensor could be an appropriate tool for such applications; however, such a subsoil measurement tool does not exist. One major reason for this is that, when penetrating compacted subsoil, high penetration forces (including friction) act on the penetrating thin rod (diameter 1 cm). The development of a tractor-mounted subsoil penetrometer for depths up to 2 m is described in this study. An ASABE standard cone is implemented, which can access heavy compacted layers. The rod, which includes wires for embedding an FDI moisture sensor in the cone tip, is covered by a protection tube. The penetration resistance measurement can be performed without being influenced by shaft friction. The rod, along with the sensor, is implemented in a tower that can be shifted laterally and can take probes in a single line without moving the tractor. To confirm the quality of the developed subsoil penetrometer, a suitable evaluation method is presented. Typical arable soil (loamy silt) was filled in boxes and compacted homogeneously using a hydraulic stamp so that different setups of the penetrometer could be compared and evaluated. The evaluation concludes that the distance between the free cone tip and the protection shaft should be at least 10 cm to measure the penetration resistance of soil without being influenced by the protection tube. Furthermore, the developed penetrometer has sufficient stability and precision for accessing subsoil. In field trials, the subsoil penetrometer was compared with a standard penetrometer and has proved its suitability.

## 1. Introduction

Strong soil compaction (SC) can be a major concern in soil degradation, especially in regions with a high mechanization level. Over the last few decades, the weights of tractors and other agricultural machinery have continuously increased. In order to counteract the ground load caused by heavier weights, the tire dimensions were significantly enlarged and the tire pressure significantly lowered. Although the tires of this machinery have been improved by increasing dimensions and decreasing inflation pressure, vertical forces on soil by the increased weight cannot be compensated in soil when pathing soil under unsuitable conditions. In contrast to topsoil, harmful compactions in subsoil are difficult to eliminate.

SC has multiple negative effects on soil functions [1]. Among others, machine traffic in the field is an issue of SC, but it is a precondition for field operations [2].

Subsoil is described as a large nutrient stock of phosphorus [3] and nitrogen [4]. Subsoil retains water in drought phases [5] and stores carbon [6]. Due to climate change and scarcity of resources, many scientific activities dealing with subsoil require sensors to access subsoil conditions and properties. Tillage is an operation restricted to the topsoil layer aiming predominantly at loosening the soil and preparing the seed bed. However, plant growth is influenced by topsoil as well as by subsoil. Because subsoil is inaccessible for mechanical treatments, permanent SC in this area is critical. Detailed information about subsoil is a crucial condition for future progress in crop farming.

Cone penetrometers are appropriate instruments for measuring penetration resistance known as the cone index [7]. Despite the significant influence of soil water content (SWC) and soil composition on the cone index, it is widely used because of its practicability and the lack of other more precise and reliable methods [8]. Theoretical methods predict the relationship between SWC and soil density (SD) using linear, polynomial, and exponential functions, but they require experimental work to be validated [2,9,10]. For assessing SC and SD, experimental methods are still preferred for evaluating mechanical soil conditions.

The development of deep soil penetrometers (DSP) is hampered by expected high penetration forces and unknown subsoil conditions (hard pens and stones), and a straight vertical movement of the penetrometer lance into the soil with a constant speed has to be ensured. Many penetrometer devices have been used and improved for different purposes. Generally, they differ in the direction of movement (vertical vs. horizontal) and the method of driving the cone into the soil [11]. Horizontal penetrometers are driven by tractors on agricultural soils. They are suitable for mapping signals when used in parallel paths across the entire field to record soil resistance data from one or more levels in depth. Andrade et al. [12] designed a horizontal penetrometer with eight cutting edges to a depth of 61 cm and used it in the field at a track distance of 1.5 m. For mapping soil conditions on the go, at a depth of 15–20 cm, [11] introduced a cone-type horizontal penetrometer equipped with an impedance sensor for SWC. The rapid response through the SWC sensor signal enabled the penetrometer to have an operation speed in a range of 0.3–1.5 ms^−1^. Hammer penetrometers use the potential energy of mass acting on the stopper. The penetration movement of the cone is correlated with SD. Sun et al. [13] compared the dynamic hammer type with a continuously moving cone penetrometer. They found that the energy-based results of a constantly driven penetrometer are overestimated compared with the hammer penetrometer. This comparison was based on a penetration depth of up to 60 cm, and no comment was given on the suitable use of penetrometer types at deeper soil levels. Continuously penetrating vertical penetrometers can be driven hydraulically or by electrical motors, but the movement through the soil must be at a constant speed following the ASAE Standard. Byun et al. [14] developed a penetrometer to access soil below strongly compacted layers. A solid tube comprising a smaller rod, including the cone, was used to characterize railway substructures. After penetrating the upper layer using a hammer, the inner cone moves out of the tube and penetrates the soil through an electrical drive.

To extrapolate from penetration resistance to soil strength or SC, the effect of soil type and SWC needs to be considered. In 1982, Ayers and Perumpel [15] stated the fundamental relationship between the cone index, soil type, and SWC. The outcome of this study gives evidence that the results of the cone index are only valid when SWC is considered. There are many proposals to measure penetration resistance and SWC simultaneously. Topp et al. [16] applied a time-domain transmissiometry probe to sense SWC in a combined instrument with a pair of wires helically surrounding the connecting rod next to the cone tip. Hummel et al. [1] applied an optical approach by implementing a sapphire lens in the cone. A fiber bundle connects the mirror’s transmitting signals to the top end of the penetrometer’s connecting rod. However, the prediction accuracy was low. Vaz and Hopmans [2] used a time-domain reflectometry (TDR) probe consisting of two parallel copper wires wrapped around a PVC cylindrical shaft directly above the cone of a hammer penetrometer. This penetrometer gives time between the impacts to compile the TDR signal to measure SWC after each penetration step.

To overcome the interruption of penetration for SWC measurements, a fast reading of SWC is required. Sun et al. [11] evaluated a penetrometer with continuous penetration, employing an embedded frequency-domain sensor in the cone tip. The tip and a metal ring in the cylindrical shaft of the connecting rod are the electrodes of the capacitive sensor system. The response time of the FDR is less than 1 s, which allows an almost continuous SWC measurement during cone penetration.

To learn about the subsoil conditions up to 2 m of depth, a vertical penetrometer had to be designed with three requirements: (I) the cone dimensions had to comply with the ASAE standard, (II) the mechanical design should admit the implementation of sensors in the cone tip, and (III) the measurements of penetration resistance should not be influenced by shaft friction caused by upper soil. Other specifications were to ease the operation to allow multiple repetitions in the field. Condition (I) results in specific demand on the mechanical design of the penetrometer, as the standard cone determines the diameter of the rod, which hampers transducing elevated forces necessary to penetrate deep soil levels. Condition (II) is related to the rod dimension as well, because connecting sensors in the cone tip cables pass inside the rod. Condition (III) also results in a specific demand on the mechanical design of the penetrometer.

The aim of the study is to introduce an innovative deep soil penetrometer (DSP) characterized by maintaining the dimensions of conventional penetrometers standardized by ASABE. Apart from explaining the design, a test device to define the interaction between the penetration forces at the cone and the friction forces prevented by an outer protection tube from the inner connecting rod was developed. The protection tube must not influence the measurement results at the cone tip. This test set was used to define the free length between the cone and bottom end of the protection tube. Finally, in a comparative field experiment, the DSP, along with a conventional penetrometer, was applied in the operation range of the latter.

## 2. Materials and Methods

The development of a subsoil penetrometer was performed through a construction methodical course of action. The first step was to create a list of requirements, which occurred during the conception phase and analysis of the state of the art.

The next step was the design of the penetrometer according to the demands and expected forces. Finally, an evaluation method had to be selected and used to describe the quality of the development. For calibration and evaluation, silty soil (14.0% sand, 83.0% silt, and 3.0% clay) was used. It represents a typical fertile soil for plant production in Germany. The gravimetric water content was approximately 12%.

**Figure 1:**
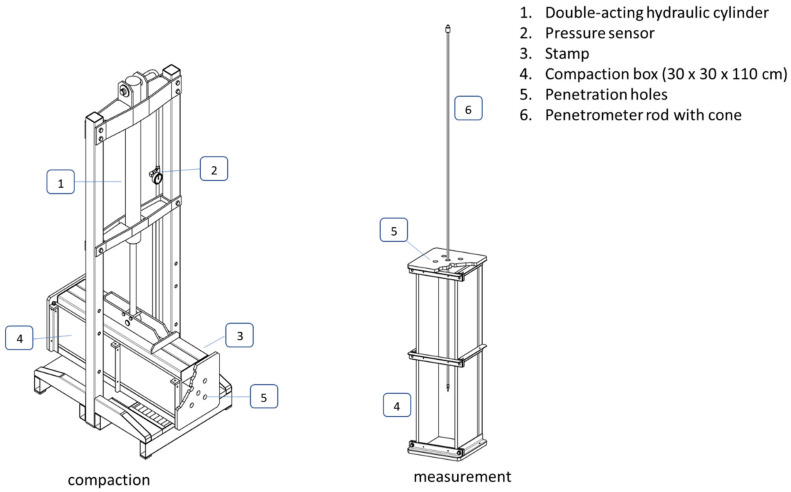
Hydraulic compactor with stamp for preparing compaction boxes (**left**) and box in an upright position for subsoil penetrometer calibration (**right**).

For this evaluation, a suitable test setup was laid out. The soil samples were compacted using compaction boxes (Figure 1, w = 30 cm, h = 30 cm, and l = 110 cm) and a hydraulic compactor (1) with a stamp (3). The compaction boxes were placed horizontally under a hydraulic press. Loose soil was evenly filled in 5 cm thick layers and compacted with a hydraulically loaded ram. This process was repeated 6 times before the box was closed with a clamping lid. The box was welded with thick-walled square tubes. The dimension corresponds approximately to the open side surface of the box. It can be assumed that the pressure distribution under the punch is homogeneous. The desired contact surface pressure could be set via a pressure valve.

In each sampling box, 6 sampling rings were placed to measure real soil density and moisture content as a reference.

After filling and compressing, the box was set up vertically and deployed for the calibration of the lance. Applying the hydraulic system with a pressure of 20 hPa resulted in a contact surface pressure of 60 N cm^−2^ and a homogeneous soil bulk density of 1.2 g cm^−3^. Four sample plunge cylinders were embedded in the box to determine the bulk density and water content.

To study the impact of the protection tube on cone resistance, five distances (l_free_ = 0, 5, 10, 15, and 20 cm) and one variant without a protection tube were installed. Each test was conducted with constant bulk density and water content and the same soil type with five repetitions.

To analyze the influence of l_free_, five penetrations with the corresponding setup were performed. To exclude the border conditions of the box, the force readings between the depths of 0–20 cm and 100–110 cm were disregarded, and the remaining range of 80 cm was considered for analysis. For comparison of the variants, the Scheffé-Test at a significance level of 5% was used.

For calibration experiments, the box was placed upright. Five holes (5) on the top of the box were used to penetrate the soil at defined positions.

In addition to the development of a new subsoil penetrometer, field measurements were conducted to compare the new subsoil penetrometer with a conventional penetrometer. For this purpose, 240 sample measurements were conducted on a typical loess site in the Rhineland (50,6355529° N 6,9765798° E), Germany (para broom soil), distributed over an area of 4 ha. The penetration signals were compared up to depths of 70 cm. The range of 70 cm results from the fact that conventional penetrometers are not suitable for deeper measurements. The soil moisture was in the range of approximately 21 vol.%. To avoid mutual influences, as well as to ensure comparability, the penetrations of both penetrometers were positioned 50 cm next to each other. The resolution in depth was 1 cm. For both penetrometer and each centimeter an average cone index and its standard deviation was calculated and compared.

## 3. Result—DSP Layout

### 3.1. Concept of the Penetration Unit

**Figure 2:**
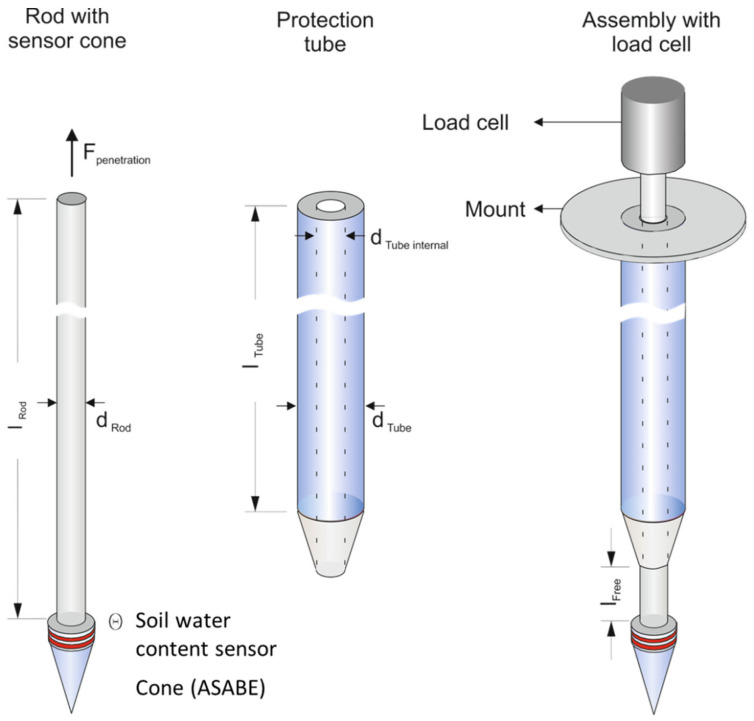
Design of the penetrometer lance for subsoil measurement, including rod with cone and sensor with two electrodes (**left**), the protection tube (**middle**), and the assembled penetrometer with load cell (**right**).

To reach the target depth of 2 m, a penetration depth of a rod length (lRod) of 2.5 m is required, which bends and even buckles when hard pens need to be penetrated. To avoid damage to the connecting rod spring steel, as specified in DIN 17223 [17], was chosen as the material. To protect the rod from bending caused by axial forces, a coaxial tube (protection tube) was provided (Figure 2).

Parallel to the penetration unit, two electrically driven ball screws were arranged to move a slide with the penetrometer rod down at a constant speed (ASABE standard EP542, v = 30 mm s^−^^1^).

The forces of the inner part (cone and rod) were measured independently of the protection tube to exclude effects due to soil friction. The cone dimensions (diameter 10 mm and tip angle 30°) were defined according to ASABE Standard S313.3 [18]. For the inner rod, a tube was chosen to admit the passing of cables for any wiring sensors embedded in the cone tip. As proposed by Sun [11], this option is provided to implement an impedance probe for SWC measurement. With this setup with two electrodes above the cone, soil penetration resistance can be determined with respect to SWC and SD.

The suitable distance (see Figure 2, l_free_) between the cone and the protection tube was determined in experiments. A large distance decreases the protection function of the rod; in contrast, a short distance can cause force impacts on the cone by the surrounding soil.

### 3.2. Layout and Specification of the DSP Frame

**Figure 3:**
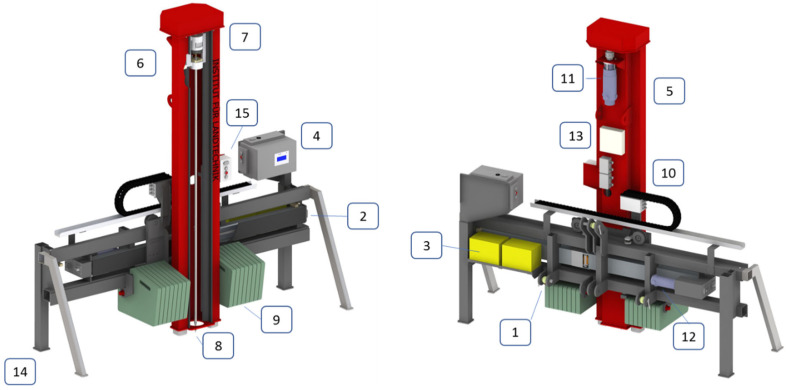
Schematic illustration of the DSP in front and rear views. 1. Tractor 3 point linkage, 2. transversal adjustment (2 m) with ball screw drive, 3. power supply, 4. control box, 5. tower with guide rails for penetrometer, 2 m down-stroke, 6. penetrometer rod with ball screw and slide, 7. load cell for tubes, 8. penetrometer tip, 9. counter balances (16 × 40 kg), 10. amplifier, 11. electrical drive for penetrometer rod, 12. electrical drive for tower movement, 13. drive controller, 14. supply pillar, 15. control panel.

The design of the subsoil penetrometer is shown in Figure 3. The subsoil penetrometer is attached to a tractor by a frame (1) with two lateral pillars (14). A plane ball screw with an electrical drive (6) is employed for the vertical movement of the tower (5) with the penetration unit (3). This feature allows multiple penetrations in one path of the carrier vehicle. Thus, SC profiles in two dimensions can be easily assigned. Penetration resistance up to depths of 2 m requires counter forces, which were supplied by manually mountable weights (640 kg) (9) and by fixing the frame to a tractor as well (12). By the adjustment of the pillar length and hydraulic top link, a penetration angle of 90° can be positioned.

The entire implementation comprises a frame with horizontal (12) and vertical (11) drives with electrical accumulators (3), summing up to 2000 kg. A tractor was chosen as the carrier vehicle, which allows for a wide range of operations, even under wet soil conditions and rough soil reliefs.

### 3.3. Data Acquisition and Processing

Penetration resistance is equivalent to the cone index, which is recorded by a load cell. The signal is amplified and digitalized by an A/D converter. The SWC measurement involves the hardware described by Sun [19].

The position of the cone is determined by two axes. The depth and lateral position are recorded by incremental counters next to the ball screw drives. A microcontroller transforms signals using software filters. The resolution in depth is defined in steps of 10 mm. The resolution of the SWC sensor is less than 1 vol.%. For each step in depth, the force and water content values are filtered, averaged, and stored as strings in a file (CSV format). For each lateral shift, a new file is created.

These files are recorded on an SD card or sent to a notebook via a USB port. In this case, the data are displayed in path-force- and path-water-content diagrams.

### 3.4. Evaluation of Experimental Setup

**Figure 4:**
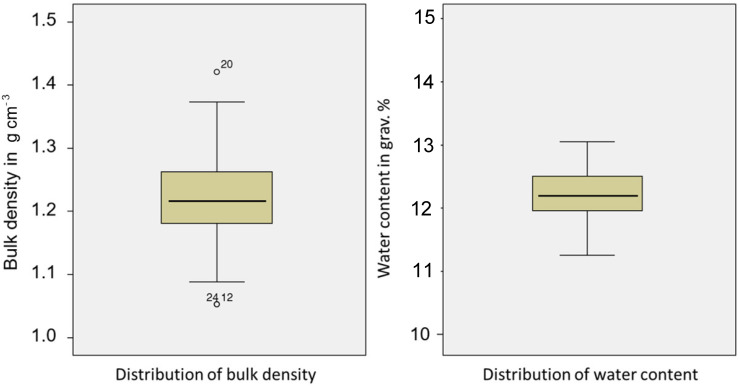
Bulk density and SWC (silty soil) in the sample boxes to optimize the penetrometer cone tip distance to the protection tube. (number of samples = 28).

The quality of soil preparation in the compaction boxes is fundamental for the application of the DSP in field experiments. Figure 4 shows the homogeneity of bulk density and water content using box plots. The randomized sample size is 28 (7 trials and 5 repetitions).

The homogeneity of soil in the boxes is evident by the average gravimetric water content of 12.20% with low scatter (SD = 0.40%) and by the bulk density with an average value of 1.21 g cm^−3^ with an SD of 0.08 g cm^−3^. The penetrometer evaluation was performed on the basis of these results.

### 3.5. Evaluation of Subsoil Penetrometer

**Figure 5:**
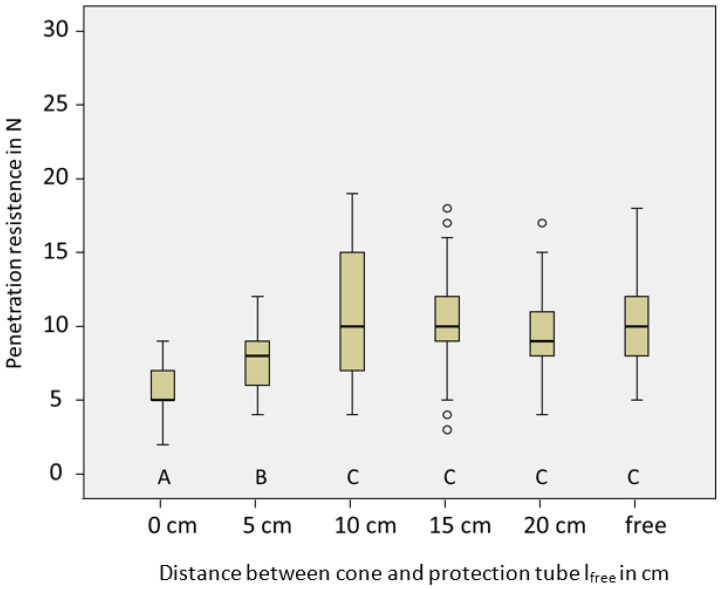
Penetration resistance (N) and standard deviation for different protection levels of the rod (l_free_) in silty soil with gravimetric water content of 12%. A, B, and C are markers for different homogeneous subgroups at a significance level of 5%.

In Figure 5, the penetration resistance (N) and standard deviation for different protection levels of the rod (l_free_) in silty soil with gravimetric water content of 12% are shown. A, B, and C are markers for different homogeneous subgroups at a significance level of 5%.

In Figure 5, the median of the penetration resistance with its scatter is displayed for all variants. The median force increased from approximately 5 N (l_free_ = 0 cm) to approximately 10 N (l_free_ = 10 cm). No significant differences were outlined for the variants “10 cm,” “15 cm,” and “20 cm” compared with the variant without the protection tube.

We conclude that for distances longer than 10 cm, the protection tube has no verifiable influence on the force affecting the cone.

### 3.6. Field Application and Comparison with a Conventional Penetrometer

**Figure 6:**
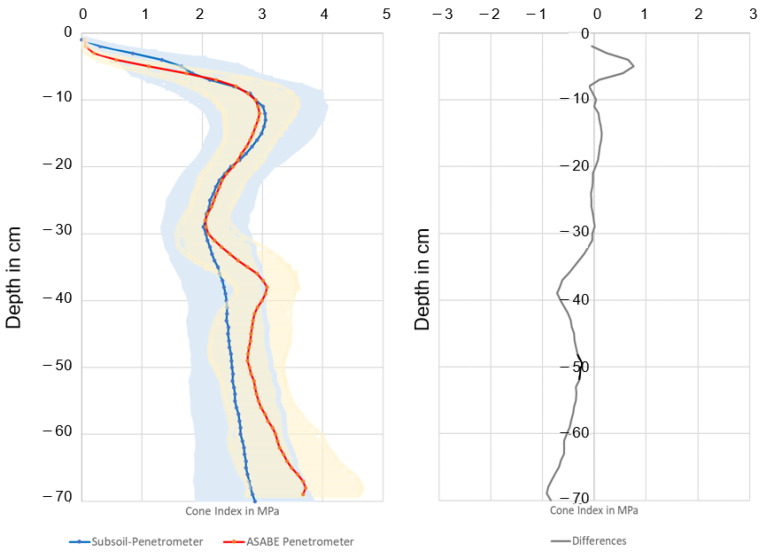
Comparison of the cone index and respective standard deviation of the SSP and a conventional penetrometer (accordant with ASABE standards) on a field (*n* = 240) (**left**), and the differences in cone index (**right**).

Figure 6 summarizes the mean values of all penetrations (displayed by color-coded lines) with a depth resolution of 1 cm. The adjacent colored areas outline the respective standard deviation. The progressions are parallel. In the upper level up to approximately 5 cm, slight deviations can be observed. In this range, the cone pressure is higher on the DSP. Up to 30 cm, the curves appear similar. Below 30 cm, the penetration forces of conventional penetrometers are higher.

## 4. Discussion

A subsoil penetrometer for accessing soil penetration resistance up to 2 m depths was developed. The tractor-mounted implement conforms to the ASABE standard. The cone index can be determined with a spatial resolution of 1 cm in depth and a variable horizontal shift. The electrical drive maintains the penetrometer at a constant speed. A defined penetration speed of the cone is ensured. The most significant component of the novel subsoil penetrometer is the protection tube, which serves as a shield for the thin penetrometer rod and prevents its destruction by high forces.

To test the accuracy of the penetrometer a suitable method for creating artificial SC was employed. This method was used for the subsequent studies.

An essential question is how to quantify the influence of the outer tube on the penetration force acting on the cone. While penetrating the soil, the tube may also cause compaction or loosening effects on the soil next to the cone, resulting in falsifications. Therefore, different distances between the cone tip and protection tube, or rather without the tube, were tested under constant soil conditions. The forces of the variants “0 cm” and “5 cm” were significantly lower than those of the other variants. The tube in this position should have a loosening effect on the soil surrounding the cone tip. For distances longer than 10 cm, no significant deviation in penetration resistance was observed. The recommendation for the application of the SSP is the l_free_ range of 10 cm because of the accurate resistance determination and the high stability of the shaft.

The SSP shows slightly higher penetration resistances in the upper centimeters than the standard penetrometer. Generally, this layer is mostly loose, and soil aggregates/clods are easily moved aside. Effective measurements in this layer are difficult, as described by Uppenkamp [19] in 1986. He developed a seedbed penetrometer for accessing the topsoil, employing a broader and flatter cone to reduce the horizontal displacement effects on the vertical forces. Considering these outcomes with respect to SSP, a wider protection tube may have a similar effect and can be judged as an advantage.

From a depth of 30 cm, the penetration resistance of the conventional penetrometer increased significantly. One reason for this could be the material of the lance [17] and the lance guide. If the lance bends, frictional forces arise on its lateral surface touching the surrounding soil, which affects the vertical forces. The protective tube of the DSP and the separate recording of the frictional forces exclude this influence.

Up to a depth of 30 cm, both curves are very similar.

## 5. Conclusions

The literature review reveals a lack of a penetrometer for subsoil measurements, which can be explained by the more difficult conditions for accessing subsoil than topsoil. On the one hand, forces acting on the cone tip and friction forces are high; on the other hand, high measurement accuracy is required.

A new penetrometer to access subsoil conditions at depths of up to 2 m was developed and tested successfully. The cone complies with the ASABE standard, and its design is robust to withstand high forces in deeper soil layers. The fundamental component is a protection tube, which stabilizes the penetrometer shaft and prevents friction forces from the shaft. The distance between the cone and the protection tube should be at least 10 cm to avoid disturbances from the protection tube on the soil underneath, where the cone forces are recorded. The layout of the penetration unit enables the embedding of an SWC sensor in the cone tip.

In addition, a suitable method for calibrating long-shafted penetrometers such as the developed subsoil penetrometer is presented.

The field tests underline the suitability of the penetrometer even for shallow depths. The penetrometer can also be used for measurements in topsoil as for subsoil.

The described technological approach can be a useful tool for scientific research on subsoil properties and functions.

## Data Availability

Not applicable.
